# Editorial: Iron dysregulation, oxidative stress, and inflammation: mechanisms and therapeutic insights

**DOI:** 10.3389/fmolb.2026.1852346

**Published:** 2026-06-08

**Authors:** Antimo Cutone, Giusi Ianiro, Claudia Fiorillo

**Affiliations:** 1 Department of Biosciences and Territory, University of Molise, Pesche, Italy; 2 Department of Experimental and Clinical Biomedical Sciences “Mario Serio”, University of Firenze, Florence, Italy

**Keywords:** inflammation, iron, oxidative stress, reactive oxygen species (ROS), therapy

## Introduction

Iron homeostasis is critical for cellular survival, acting as an essential cofactor in DNA synthesis, myelination, and mitochondrial respiration. However, its redox activity also renders iron intrinsically hazardous: when the balance of the labile iron pool is disrupted, it catalyzes the formation of reactive oxygen species (ROS) and lipid hydroperoxides, triggering a self-amplifying cycle of oxidative stress and inflammation.

This Research Topic in *Frontiers in Molecular Biosciences* comprises five studies, including three original research articles and two review articles, collectively highlighting a conceptual shift in the field from isolated observations to an integrated framework in which iron dysregulation acts as a “pathogenic amplifier,” transforming localized metabolic disturbances into progressive and systemic damage. By spanning acute injury and chronic disease contexts, these contributions provide a coherent molecular perspective on ferroptosis-driven pathology.

## Acute conditions

In acute pathological settings, ferroptosis emerges as a central driver of tissue injury.

In ischemic stroke, the review by Long et al. describes ferroptosis as a coordinated metabolic collapse regulated by the GPX4/ACSL4 axis and driven by iron-catalyzed Fenton reactions. The authors further identify regulatory pathways such as USP14/GPX4 and USP14/NCOA4, linking ferritinophagy to iron release and oxidative damage amplification. Importantly, the identification of FABP5 as a specific biomarker of early ferroptosis provides a clinically relevant tool to define therapeutic windows and guide targeted interventions, including ferrostatin-1 and liproxstatin-1.

A parallel mechanism is observed in acute lung injury. Yang et al. demonstrate that the flavonoid Farrerol exerts protective effects by stabilizing RUNX1 and upregulating SLC7A11, thereby enhancing glutathione synthesis and suppressing lipid peroxidation. Their findings show significant attenuation of pulmonary edema and tissue damage, highlighting ferroptosis inhibition as a promising therapeutic strategy in acute inflammatory conditions.

## Chronic conditions

In chronic diseases, iron dysregulation contributes to progressive and system-wide dysfunction.

In patients undergoing peritoneal dialysis, Li et al. provide original evidence linking regional brain iron accumulation to cognitive impairment. Using QSM imaging, the study reveals an inverted U-shaped relationship between serum ferritin and cognitive performance, with a critical threshold at 258.4 μg/L. Notably, iron deposition in the amygdala correlates with deficits in attention, working memory, and emotional regulation, independently of peripheral iron markers, suggesting a decoupling between systemic and central iron homeostasis.

Neurodegenerative processes are further explored in Parkinson’s disease. Su et al. show that probiotic supplementation reduces iron deposition in the substantia nigra and improves motor function in a rat model. These effects are mediated through the gut–brain axis, involving modulation of microbiota, enhancement of antioxidant defenses, and preservation of blood–brain barrier integrity. Importantly, ESWAN imaging is validated as a reliable non-invasive tool for monitoring brain iron levels.

In oncological settings, ferroptosis plays a dual role within the tumor microenvironment. The review by Han et al. highlights how glioblastoma cells exploit iron metabolism, lipid remodeling, and antioxidant defenses to balance proliferation and ferroptotic vulnerability. Ferroptosis can promote antitumor immunity through damage-associated molecular patterns, yet also contributes to immunosuppression by favoring regulatory T cells and M2 macrophages. Emerging strategies—including nanodelivery systems and combination therapies with immune checkpoint inhibitors—aim to exploit this balance for therapeutic benefit.

## Concluding remarks

Taken together, these studies converge on a unifying concept: regardless of whether the initiating event is acute injury or chronic disease, iron dysregulation represents a common mechanistic denominator. The expansion of the labile iron pool, the accumulation of lipid peroxidation products, and the activation of inflammatory cascades define a shared pathogenic axis across diverse conditions.

The integration of these findings supports the emerging paradigm of “precision iron medicine,” combining advanced imaging techniques such as QSM and ESWAN with molecular biomarkers like FABP5. However, key challenges remain, including the identification of optimal therapeutic windows, the development of targeted delivery systems capable of crossing biological barriers, and the need for large-scale clinical validation.

Future strategies will likely rely on multimodal approaches integrating anti-ferroptotic agents, metabolic interventions, and microbiota modulation. Mastering the regulation of the iron–oxidation–inflammation axis may ultimately offer the possibility not only to slow, but potentially to halt, the progression of both neurodegenerative and systemic diseases.

